# Dissociation of C-Reactive Protein Localizes and Amplifies Inflammation: Evidence for a Direct Biological Role of C-Reactive Protein and Its Conformational Changes

**DOI:** 10.3389/fimmu.2018.01351

**Published:** 2018-06-12

**Authors:** James D. McFadyen, Jurij Kiefer, David Braig, Julia Loseff-Silver, Lawrence A. Potempa, Steffen Ulrich Eisenhardt, Karlheinz Peter

**Affiliations:** ^1^Atherothrombosis and Vascular Biology Laboratory, Baker Heart and Diabetes Institute, Melbourne, VIC, Australia; ^2^Department of Clinical Haematology, The Alfred Hospital, Melbourne, VIC, Australia; ^3^Australian Centre for Blood Diseases, Monash University, Melbourne, VIC, Australia; ^4^Department of Plastic and Hand Surgery, University of Freiburg Medical Centre, Medical Faculty of the University of Freiburg, Freiburg, Germany; ^5^College of Pharmacy, Roosevelt University, Schaumburg, IL, United States; ^6^Heart Centre, The Alfred Hospital, Melbourne, VIC, Australia; ^7^Department of Immunology, Monash University, Melbourne, VIC, Australia

**Keywords:** inflammation, C-reactive protein, cardiovascular diseases, ischemia/reperfusion, Alzheimer disease

## Abstract

C-reactive protein (CRP) is a member of the pentraxin superfamily that is widely recognized as a marker of inflammatory reactions and cardiovascular risk in humans. Recently, a growing body of data is emerging, which demonstrates that CRP is not only a marker of inflammation but also acts as a direct mediator of inflammatory reactions and the innate immune response. Here, we critically review the various lines of evidence supporting the concept of a pro-inflammatory “CRP system.” The CRP system consists of a functionally inert circulating pentameric form (pCRP), which is transformed to its highly pro-inflammatory structural isoforms, pCRP* and ultimately to monomeric CRP (mCRP). While retaining an overall pentameric structure, pCRP* is structurally more relaxed than pCRP, thus exposing neoepitopes important for immune activation and complement fixation. Thereby, pCRP* shares its pro-inflammatory properties with the fully dissociated structural isoform mCRP. The dissociation of pCRP into its pro-inflammatory structural isoforms and thus activation of the CRP system occur on necrotic, apoptotic, and ischemic cells, regular β-sheet structures such as β-amyloid, the membranes of activated cells (e.g., platelets, monocytes, and endothelial cells), and/or the surface of microparticles, the latter by binding to phosphocholine. Both pCRP* and mCRP can cause activation of platelets, leukocytes, endothelial cells, and complement. The localization and deposition of these pro-inflammatory structural isoforms of CRP in inflamed tissue appear to be important mediators for a range of clinical conditions, including ischemia/reperfusion (I/R) injury of various organs, cardiovascular disease, transplant rejection, Alzheimer’s disease, and age-related macular degeneration. These findings provide the impetus to tackle the vexing problem of innate immunity response by targeting CRP. Understanding the “activation process” of CRP will also likely allow the development of novel anti-inflammatory drugs, thereby providing potential new immunomodulatory therapeutics in a broad range of inflammatory diseases.

## Introduction

C-reactive protein (CRP) is a member of the pentraxin superfamily and was first discovered in 1930 by Tillett and Francis (1). Indeed, the first characterization of this protein was based on the initial observation that a distinct third fraction identified from the sera of patients with pneumococcus infection could precipitate the “C” polysaccharide derived from the pneumococcus cell wall. Subsequently, Avery and McCarty described CRP as an acute phase reactant after demonstrating that CRP levels were elevated in patients with a range of inflammatory conditions (2–4). Some 40 years after the original description of CRP, phosphocholine (PC) was shown to be the specific ligand for CRP binding within the pneumococcal cell wall (5). Today, CRP is widely used in the clinic as a marker of inflammation (6).

However, importantly, there is now a large body of evidence from prospective clinical trials that CRP levels may serve as a predictor of cardiovascular events, thus bringing the biological role of CRP into focus (7–9). This review discusses how insights gained into the different structural isoforms of CRP have led to a greater appreciation of its pro-inflammatory and prothrombotic role, which is relevant to a broad range of disease states.

## CRP Structure and Function

C-reactive protein is predominantly synthesized by the liver as a pentamer composed of five identical, non-covalently linked 23 kD protomers—each one folded into two antiparallel β-sheets with a “jelly roll” topology (10, 11). Each protomer has a binding face with a PC-binding site, which binds apoptotic cell membranes and bacterial cell walls (12). The two key residues of the hydrophobic binding pocket essential for mediating PC binding are Phe-66 and Glu-81. Phe-66 regulates hydrophobic interactions with the methyl group of PC, while Glu-81 interacts with the positively charged nitrogen (12). The opposite face of the binding face, known as the effector face, binds the globular domain of the complement factor 1q (C1q) and Fc gamma receptors, thus providing a mechanism to activate the innate immune system (13). However, the location of these binding sites on the pentameric form of CRP (pCRP) appears to be cryptic, thus supporting the concept that pCRP does not possess intrinsic pro-inflammatory properties.

## Conformational Change of CRP Creates Highly Pro-Inflammatory Molecules

pCRP dissociates into monomeric CRP (mCRP) after exposure to heat, urea, or an acidic microenvironment (14, 15). The dissociation of pCRP into its subunits exposes a range of neoepitopes that are likely to account for the distinct pro-inflammatory function of mCRP (16, 17) (see below). More recently, the *in vivo* generation and consequences of mCRP production have begun to be elucidated. Indeed, we and others have demonstrated that pCRP can be dissociated by calcium-dependent binding to liposomes and cell membranes (16–18). Activated platelets (19), endothelial cells (20), and monocytes (17) may provide the requisite PC-binding sites to facilitate the dissociation of pCRP to mCRP. This process is phospholipase A2 dependent since PC exposure and hence pCRP dissociation are dependent upon PLA2 generation (20).

The dissociation of pCRP to mCRP also produces marked changes in the solubility of the respective structural isoforms. While pCRP is soluble, the dissociation produces a shift from a predominantly β-sheet tertiary protein conformation to protomers with an α-helical tertiary structure and the exposure of previously cryptic interprotomer contacts (17). As such, mCRP has little solubility, and this led to the concept that this isoform was predominantly a tissue-bound form of CRP.

## Identification of the Pro-Inflammatory pCRP* Structural Isoform

Very recent work from our group has provided additional new insights into the mechanism of pCRP dissociation. The conformational change of pCRP that occurs on the PC-rich and highly curved MP membranes (derived from activated monocytes, platelets, or endothelial cells) initiates a spatial separation of the five CRP monomers in relation to each other (17). Therefore, MPs with bound, dissociated pCRP* act as transport vehicles of circulating pCRP* to distant sites. In this regard, microparticles with bound dissociated CRP can be detected in patients with acute coronary syndromes (21). In contrast, mCRP appears to be rapidly cleared from the circulation due to its unfolded and more disordered state. Moreover, while most studies have investigated the presence of mCRP as the dissociated structural isoform in inflamed tissue, we have shown that pCRP* is the dominant isoform in injured tissue, including inflamed human muscle, burn wounds, and human atherosclerotic plaques.

**Figure 1:**
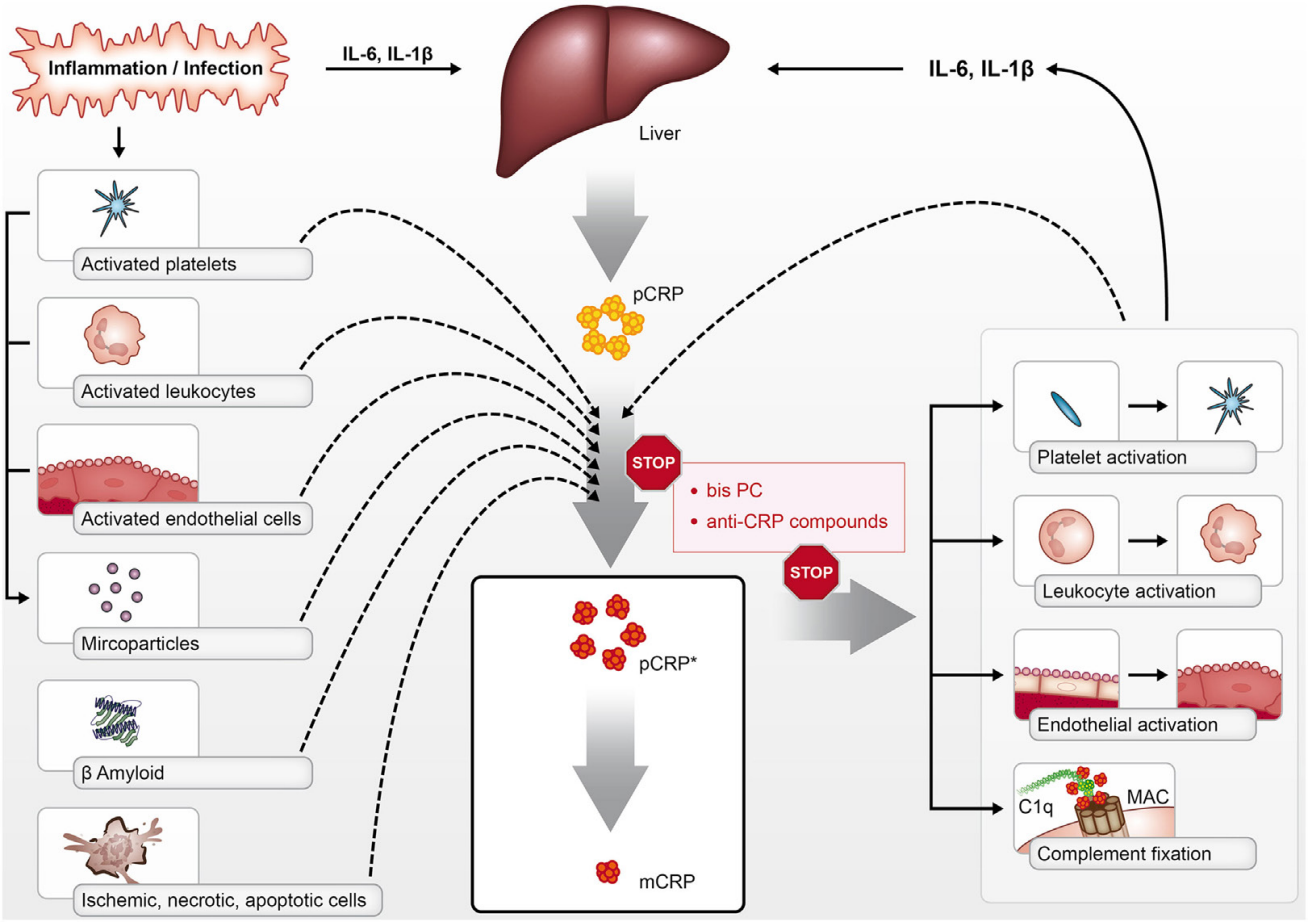
pCRP is produced by the liver in the context of inflammation and infection in response to cytokines such as interleukin 6 (IL-6) and IL-1β. pCRP can be dissociated into its pro-inflammatory structural isoforms [pCRP* and monomeric CRP (mCRP)] on the surface of activated platelets, leukocytes, endothelial cells, phosphocholine-rich MPs, dying cells, and beta-amyloid. pCRP* and mCRP can activate platelets, leukocytes, and endothelial cells, in addition to activating complement *via* C1q binding. The pro-inflammatory effects of pCRP*/mCRP can be inhibited with compounds such as bisPC that inhibit the dissociation of pCRP and block mCRP effector binding.

Crucially, both pCRP* and mCRP have pro-inflammatory functions; however, the structural isoform-specific antibodies commonly used to distinguish pCRP from dissociated isoforms cannot differentiate between mCRP and pCRP*. Although still existent as a pentamer, this intermediate form—termed pCRP*—exposes functionally active neoepitopes, which are recognized by isoform-specific antibodies and allow the binding of C1q (1, 17). These studies have led to the concept of conformation-dependent regulation of the innate response by CRP. While pCRP does not display any pro-inflammatory properties, as discussed below both mCRP and pCRP* are potent pro-inflammatory structural isoforms of CRP, which can mediate immune, inflammatory, and prothrombotic responses in a range of diseases (Figure 1).

## Location and Regulation of CRP Production

The vast majority of CRP is produced in the liver (22). While the production of CRP has also been reported in an array of other cell types including leukocytes (23), adipocytes (24), neuronal cells (25), renal cells (26), and respiratory epithelial cells (27), the extrahepatic production of CRP is not thought to influence plasma CRP concentrations significantly. In health, plasma CRP levels may be undetectable; however, in the context of inflammation, they can increase by 1,000-fold within 24–72 h (28). Thus, the CRP plasma level is widely used as a marker in reporting on the general inflammatory status of patients (29–32).

The induction of CRP expression and secretion by hepatocytes is principally regulated by interleukin 6 (IL-6) and to a lesser extent interleukin 1β (IL-1β) (33). In contrast, interferon-α, statins, and nitric oxide suppress the induction of pCRP expression by pro-inflammatory cytokines, resulting in a weak correlation of serum CRP levels with viral infections or systemic lupus (34–36). Indeed, the pro-inflammatory cytokines induce hepatic production of several acute phase proteins. This upregulation occurs *via* the transcriptional activation of the STAT3, C/EBP, and NF-κB pathways (33, 37, 38). In the context of CRP, the recruitment and activation of the C/EBP family members C/EBPβ and C/EBPα appear critical to the induction of CRP. Moreover, the STAT3 and Rel proteins bind to the proximal CRP promoter, with the ensuing interactions resulting in enhanced binding of C/EBP and thereby facilitating maximal CRP induction.

## The Pro-Inflammatory and Prothrombotic Properties of CRP

As discussed, dissociated mCRP and pCRP* are considered the pro-inflammatory components of the “CRP system.” While most work has focused on the biological effects of mCRP, pCRP* exhibits similar effects. Consistent with this, mCRP has been demonstrated to activate monocytes, thus leading to Mac-1 activation and resulting in enhanced monocyte adhesion *in vitro* (19). Moreover, mCRP-stimulated monocytes induce reactive oxygen species generation (19). While the binding of mCRP to monocytes is at least partially dependent upon the Fc gamma receptors (CD64, CD32, CD16), it also appears that lipid rafts are essential for mCRP binding since disruption of these with agents such as nystatin completely inhibits mCRP binding (39–41). In accordance with the pro-inflammatory properties of mCRP, neutrophils in response to mCRP stimulation upregulate Mac-1 binding, which correlates with an increase in neutrophil adhesion on activated endothelial cells *in vitro* (42). In addition, mCRP stimulates the production of interleukin 8 (IL-8) from neutrophils *via* intracellular peroxynitrite production (43).

Monomeric CRP can also activate endothelial cells (41, 44). Under normal conditions, the endothelium maintains a quiescent state to prevent the unwanted adhesion of platelets and leukocytes. However, upon activation, inflamed endothelium upregulates the expression of many adhesion receptors, which facilitate platelet and leukocyte interaction. Two of the main endothelial adhesion receptors, intercellular adhesion molecule 1 (ICAM-1) and vascular cell adhesion molecule 1 (VCAM-1), are upregulated by endothelial cells in response to mCRP stimulation (18, 41, 44). ICAM-1 serves as an important counter-receptor for the leukocyte integrin lymphocyte function-associated antigen 1 and thereby enhances leukocyte recruitment to sites of endothelial inflammation (45). Moreover, ICAM-1 can bind fibrinogen and thus serves as an important counter-receptor for the platelet integrin GPIIb/IIIa and thus mediates stable platelet adhesion to the inflamed endothelium (46). Likewise, VCAM-1 binds to the leukocyte integrin very late antigen 4 and serves to promote leukocyte adhesion (47). In addition, mCRP has been demonstrated to stimulate endothelial chemokine production, with both IL-8 and monocyte chemoattractant protein-1 release being increased in the context of mCRP activation (41, 44, 48).

Platelets, which mediate thrombosis and hemostasis, also have important innate immune functions (49). In this regard, mCRP has been shown to activate platelets as demonstrated by its ability to induce GPIIb/IIIa activation and alpha granule exocytosis (as measured by P-selectin expression) in a process dependent upon p38 MAPK and JNK signaling (50, 51). The receptor responsible for mediating the prothrombotic effects of mCRP on platelets has not been clearly elucidated. However, the platelet scavenger receptor CD36 plays a vital role given that CD36 inhibition blocks some of the effects of mCRP (50). Interestingly, the membrane of activated platelets appears to be an essential substrate mediating pCRP dissociation and, in this context, growing thrombi *in vitro* can dissociate CRP, thus promoting thrombus growth (50, 51).

The complement cascade is an essential arm of the immune system. In keeping with the concept that mCRP modulates the innate immune response, mCRP can bind and activate the complement system (52). mCRP and pCRP* readily bind to C1q and thus lead to robust activation of the classical complement cascade, which ultimately culminates in the formation of the membrane attack complex (17). Recent experimental work has demonstrated that the globular head of C1q can only bind to dissociated mCRP or spatially altered pCRP*, but not to pCRP, since this appears to represent the crucial structural determinant regulating complement activation (17). Given the highly conserved nature and universal presence of PC in eukaryotic cells, the ability of PC to bind CRP may serve to direct the opsonization of apoptotic and necrotic cells by the complement system, thus facilitating their clearance (28, 53, 54).

Interestingly, mCRP appears to demonstrate a dual role in regulating the complement system, since it not only serves to activate the complement cascade but also functions as a regulator of the degree of complement activation. In this regard, mCRP can also bind complement regulatory proteins such as Factor H and direct these to sites of cellular damage (55, 56). This process acts to enhance C3b inactivation, thus limiting the further generation of inflammatory products and aiding in the opsonization and subsequent clearance of damaged cells (55, 57). However, under pathological conditions, these responses may be maladaptive and promote the pathogenesis of inflammatory and immune conditions.

As will be discussed, these observations that pCRP* and mCRP can enhance innate immune responses have relevance to a range of clinical conditions and, as such, they may represent potentially novel therapeutic targets for a diverse spectrum of diseases.

## CRP in Ischemia/Reperfusion

**Figure 2:**
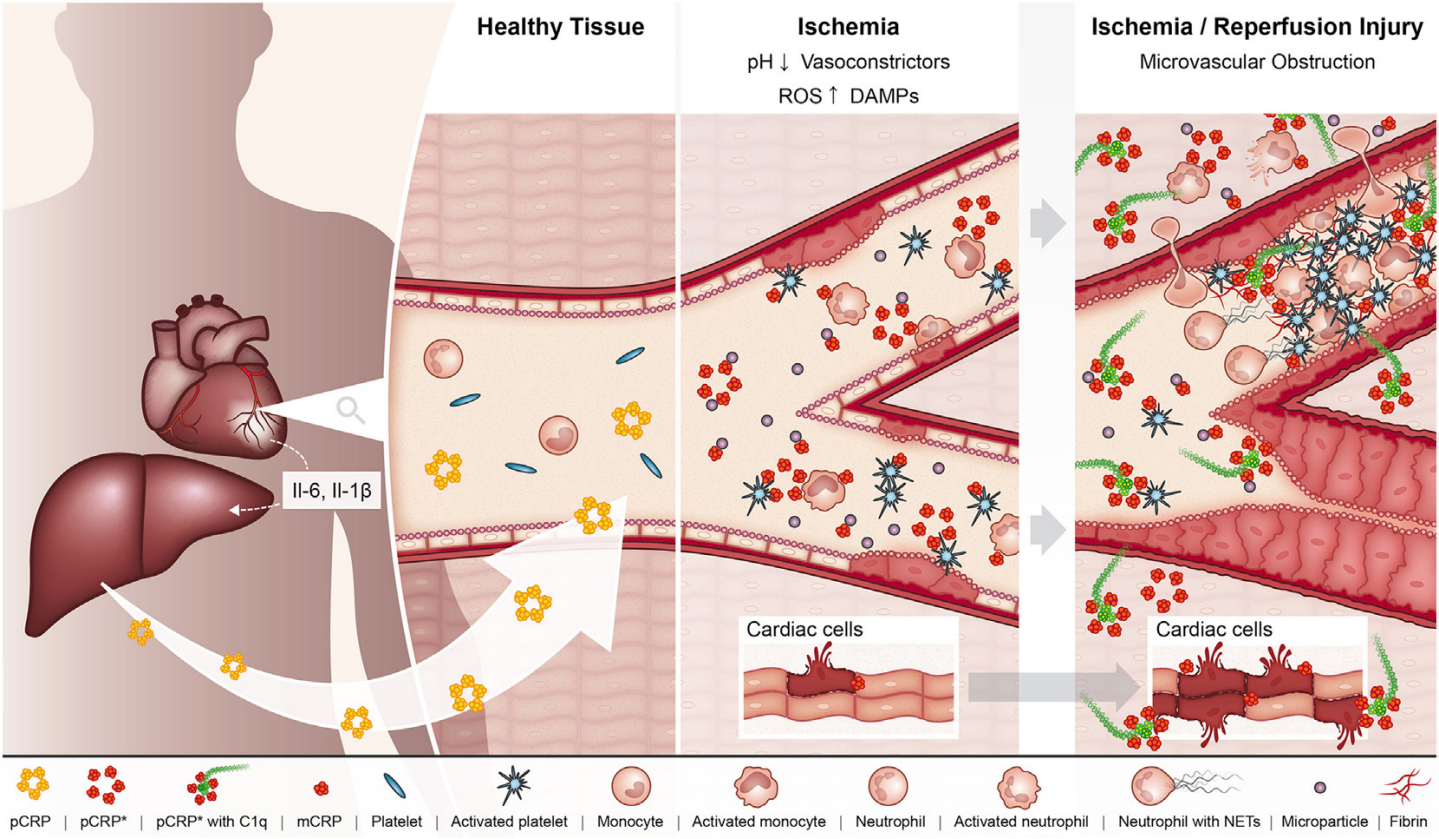
In the setting of myocardial ischemia, the hepatic production of pCRP is upregulated under the regulation of interleukin 6 (IL-6). pCRP circulates in the blood, where it can interact with MPs, activated platelets, leukocytes, and endothelial cells in the ischemic myocardium, which triggers the dissociation of pCRP to its pro-inflammatory forms pCRP* and monomeric CRP (mCRP). In turn, these pro-inflammatory structural isoforms of CRP aggravate tissue injury by promoting the adhesion of leukocytes and platelet deposition to the ischemic endothelium, ultimately resulting in the formation of microvascular thrombi and thus microvascular obstruction. In addition, pCRP*/mCRP enhances leukocyte transmigration where infiltrating leukocytes degranulate and exacerbate tissue injury. pCRP*/mCRP also activate complement, which can directly mediate tissue injury *via* formation of the membrane attack complex. These pro-inflammatory effects of CRP ultimately result in enhanced myocardial injury and impaired organ function.

Ischemia/reperfusion injury (IRI), referring to the restoration of blood flow to a previously ischemic organ, significantly contributes to morbidity and mortality in a range of clinical scenarios, including myocardial infarction and ischemic stroke (58). Indeed, experimental evidence suggests that up to 50% of the final infarct volume from myocardial infarction is due to reperfusion injury (59). One of the classic features of IRI is the initiation of a maladaptive immune response, which leads to widespread microvascular dysfunction and exacerbation of organ injury (58). A large body of data now exists demonstrating that CRP plays an active role in exacerbating IRI (Figure 2). The administration of pCRP to rats after the onset of myocardial ischemia has been shown to increase infarct volume by 40% in a complement-dependent manner (60). In accordance, pCRP administered to rats prior to the onset of cardiac reperfusion results in the marked deposition of mCRP in the infarcted myocardium, which correlates to the degree of leukocyte infiltration, apoptosis (as measured by caspase-3), and expression of IL-6 and TNF-alpha (20).

Critically, mCRP, but not pCRP, is abundant within infarcted human myocardium and also co-localizes with complement staining (20, 61). Our group has recently confirmed these observations in a rat model of IRI where rats administered pCRP displayed more severe renal tubular damage, which correlated with increased mCRP deposition, leukocyte infiltration, and caspase-3 activation after IRI (manuscript under review). Interestingly, the effects of CRP here appear primarily dependent upon facilitating endothelial–leukocyte interactions and enhancing leukocyte ROS generation in a process linked to mCRP binding to leukocyte lipid raft domains. These findings are consistent with previous work utilizing transgenic mice that express human CRP where, after renal IRI, these mice displayed more severe renal injury associated with a diversion of the monocyte/macrophage profile away from a protective M2 profile toward a “pro-inflammatory,” deleterious M1 profile in the context of IRI (62).

Studies using intravital microscopy have been used to delineate the spatiotemporal aspects of CRP conformational changes in the context of tissue inflammation. mCRP becomes deposited on apoptotic endothelial cells after LPS challenge, and this leads to enhanced leukocyte rolling, adhesion, and transmigration (20). CRP is also transported into the inflamed tissue by transmigrating leukocytes, with leukocyte infiltrates in inflamed tissue co-localizing with mCRP deposition (17, 20). These pro-inflammatory effects of CRP are principally complement-dependent, since depletion of complement *in vivo* is associated with a marked inhibition of leukocyte adhesion in the context of exogenous pCRP administration. Moreover, monocytes in rats administered exogenous pCRP in the context of LPS-induced endothelial inflammation also demonstrated increased ROS generation *in vivo*, thus demonstrating another mechanism, in addition to complement activation, by which mCRP can mediate tissue damage (20). In accordance with these *in vivo* findings, sections of human striated muscle, which have undergone IRI also demonstrated strong mCRP staining, which co-localized with monocytes/macrophages (20). Interestingly, recent findings from our group demonstrate that circulating pCRP binds to the cell membrane of LPS-activated, but not resting, monocytes *in vitro* and *in vivo* (17). Here, CRP binds to stimulated monocytes, which release pro-inflammatory microvesicles loaded with pCRP*, thus allowing binding of the complement factor C1q and ultimately causing complement activation.

The acidic pH at sites of inflammation or ischemia can also lead to exposure of CRP neoepitopes (14–16). Emphasizing the broad biological relevance of CRP deposition and dissociation in damaged tissue, CRP has also been demonstrated to co-localize with complement factor C3d and leukocytes in tissue samples from burned wounds (63). Moreover, mCRP deposition has not only been found exclusively in areas of damaged tissue co-localized with CD68-positive monocytes and complement but also correlates with the predicted burn depth (64). Complementary *in vitro* data suggest that mCRP opsonizes necrotic cells and accelerates phagocytosis by macrophages (64). These observations, combined with previous data supporting a role of complement in mediating wound healing, suggest that the role of mCRP in this context likely extends beyond its direct pro-inflammatory effects and it may promote wound scarring and contracture development.

## CRP in Allogeneic Transplantation

Allogeneic solid organ transplantation is a curative therapy for patients with end-stage organ failure. Despite improvements in immunosuppressive protocols and supportive care, close to half of all patients lose their allografts within 10 years after transplantation due to chronic graft rejection and graft dysfunction (65). Based on multiple retrospective studies of human kidney transplantation, an acute rejection episode has been shown to be associated with a significantly decreased 1-year graft survival (66, 67), in addition to being linked to adverse long-term survival and graft outcomes (68).

Allograft rejection triggered by major histocompatibility complex incompatibility is dependent on T-cell activation. However, it has recently been demonstrated that innate recognition of allogeneic non-self by the recipient’s monocytes can also initiate graft rejection (69). Importantly, it is now apparent that other factors such as IRI play a critical role in mediating the immune response to allogeneic transplantation. In solid organ transplantation, the impacts of IRI on both acute rejection and long-term allograft survival have been extensively investigated (70–72). For instance, increased rates of acute rejection episodes have been reported in experimental renal transplant models when ischemic times were prolonged (73) and large clinical trials have shown that the duration of cold ischemia time adversely correlates with allograft survival (72, 74). However, the relevance of the innate response to acute and chronic rejection in allogeneic transplantation is poorly understood.

Donor endothelial cells are the predominant target of the alloimmune response because these cells represent the first barrier to the recipient’s immune system. Thus, allograft rejection typically manifests in the microvasculature of the transplanted tissue (75). IRI generates damage-associated molecular patterns (DAMPs) such as heat shock proteins and ROS, heparin sulfate, or fibrinogen (76). DAMPs, in turn, can bind and activate toll-like receptors, which results in a strong inflammatory response and release of IL-1β, IL-6, and tumor necrosis factor, as well as CRP production (77).

With respect to the pro-inflammatory effects of CRP and its contribution to postischemic tissue injury, we have investigated the impact of CRP on innate allogeneic recognition and graft rejection in a hind limb transplantation model in rats (unpublished data). Based on our previous studies, we hypothesized that CRP-aggravated tissue damage and inflammation are associated with higher rates of acute and chronic rejection. Furthermore, we investigated the potential of inhibiting the conformational change of pCRP with bisPC as a therapeutic target for allogeneic transplantation *in vivo*. In our study, the administration of pCRP significantly accelerated clinical allograft rejection. We identified that the tissue damage-induced conformational change of pCRP led to increased systemic activation and localized transmigration of monocyte subpopulations. Most importantly, the stabilization of pCRP with bisPC abrogated its immunomodulatory effects and consequently inhibited aggravation of the acute transplant rejection (unpublished data). Thus, the inhibition of pCRP dissociation represents a promising, novel immunomodulatory therapeutic strategy in allogeneic solid organ transplantation.

## CRP in Atherosclerosis

Inflammation incites and promotes the progression of atherosclerosis (78). In this regard, the role of CRP as a biomarker reflecting cardiovascular risk has received much attention (79, 80). Supporting the concept that CRP may reflect cardiovascular risk, data from the Women’s Health Study and Physicians’ Health Study demonstrated that CRP is a strong predictor of future cardiovascular events (81, 82). Moreover, the large-scale JUPITER trial has shown that treating patients with asymptomatic elevations in CRP provides benefit in reducing the cardiovascular event rate and death (7). Most interestingly, in the recent highly publicized CANTOS trial, the extent of the reduction of CRP achieved with the anti-Il-1β antibody canakinumab correlated directly with the extent of reduction in primary endpoint events, mainly myocardial infarction (8).

However, some controversy persists regarding whether CRP is a useful predictive biomarker in cardiovascular disease and this may reflect the fact that standard assays measure total CRP, rather than explicitly measuring the pro-inflammatory monomeric structural isoform (83). Aside from acting as a potential biomarker for cardiovascular disease, there is now a wealth of experimental data suggesting a direct causative role for CRP in promoting cardiovascular disease. Indeed, vulnerable plaques have been demonstrated to release CRP, which correlated with neutrophil Mac-1 activation (84). Additionally, CRP is present within atherosclerotic plaques and here is co-localized with macrophages, complement, and oxidized LDL (85). Significantly, we have demonstrated that mCRP, but not pCRP, is present within atherosclerotic plaques and that mCRP-bearing microparticles are significantly increased in patients with acute coronary syndromes (19, 21). Importantly, mCRP, but not pCRP, is detected within infarcted human myocardium, where it co-localizes with macrophages (20). More recently, *in vivo* work has demonstrated that mCRP accumulates at sites of endothelial inflammation and this results in enhanced leukocyte adhesion and transmigration (17). Demonstrating the importance of mCRP in exacerbating tissue injury, inhibition of pCRP dissociation with bisPC has been shown to reduce infarct size in a rat model of myocardial infarction (86).

These findings support the concept that mCRP may not only be a valuable biomarker in cardiovascular disease but also directly acts to promote a deleterious inflammatory response in the context of atherogenesis and myocardial infarction.

## CRP in Alzheimer’s Disease (AD)

Alzheimer’s disease represents the leading cause of dementia (87). The underlying pathogenesis of AD appears to involve the accumulation of amyloid beta (Aβ) plaques in extracellular spaces and within the walls of the vasculature (88). The later stages of AD typically involve the accumulation of neurofibrillary tangles—comprised of Tau protein aggregates—in neurons (89). Previous studies have demonstrated that neuronal tissue from patients with AD expresses higher amounts of CRP compared to non-AD controls and that CRP staining co-localizes with senile plaques (90, 91). Recently, evidence is beginning to emerge that CRP may actively contribute to the pathogenesis of AD. Aβ plaques can dissociate pCRP *in vitro* and, in accordance with this, mCRP and C1q are observed to co-localize with Aβ plaques in human AD sections (92).

Supporting the concept that mCRP may directly contribute to AD pathogenesis, recent data demonstrate that the hippocampal injection of mCRP in a mouse model of AD induces cognitive decline and behavioral changes (93). These features correlate with structural changes, with mCRP-treated mice displaying enhanced p-Tau and p-β amyloid plaque production (93). mCRP has also been demonstrated to induce Tau filament polymerization and directly trigger potentially neurodegenerative signaling pathways in rat cortical neurons *in vitro*. Interestingly, mCRP is also abundant within the microvasculature of patients with AD and co-localizes with β amyloid and CD105 (93). This observation appears most prominent in patients with prior ischemic stroke, which raises the interesting possibility that mCRP deposition in these regions promotes dysregulated angiogenesis and, therefore, promulgates the small vessel vasculopathy characteristic of AD.

## CRP in Age-Related Macular Degeneration (AMD)

Age-related macular degeneration is a leading cause of blindness in developed countries (94). It has previously been demonstrated that elevated CRP levels serve as an independent risk factor for the development and progression of AMD (95). Recently, it was shown that mCRP plays a potentially crucial pathogenic role in patients with the high-risk CFH SNP (Y402H) (96). This SNP involving the CFH gene is considered one of the most significant genetic risk factors for AMD development (97). Indeed, levels of mCRP, but not pCRP, are increased in the choroid of patients with the high-risk CFH mutation (96). Moreover, the application of mCRP to choroidal endothelial cells *in vitro* results in enhanced endothelial cell permeability and migration, both of which are important for AMD development (96). Further supporting the notion that mCRP may promote AMD development, human choroidal tissue exhibits an altered transcriptome in response to mCRP stimulation. The expression of both ICAM-1 and carbonic anhydrase 4 (CA4) is altered at the mRNA and protein level, with mCRP resulting in increased ICAM-1 expression and decreased CA4 (96). These are important observations, given that these changes are observed in patients with AMD and are linked to the development of AMD (98, 99).

## Therapeutic Targeting of CRP

The powerful pro-inflammatory properties of pCRP* and mCRP in mediating a broad range of disease states make inhibition of CRP an attractive therapeutic strategy. In this regard, 1,6 bis-phosphocholine (bisPC) has previously been shown to ameliorate the deleterious effects of CRP in mediating myocardial IRI (20, 86). Indeed, bisPC prevents mCRP formation and deposition in infarcted myocardium in addition to preserving myocardial function in the context of IRI. bisPC abrogates the pro-inflammatory effects of CRP by binding to and preventing CRP dissociation, in addition to blocking the binding of pCRP to MPs, thus inhibiting the interactions of CRP with complement (17, 86). However, the pharmacokinetic profile of bisPC is unfavorable, given its relatively low affinity for CRP (Kd = 150 nM) and relatively short half-life of 90 min in mice (86). Therefore, there remains much interest in developing novel, more potent inhibitors of CRP with improved bioavailability.

## Perspective

Deepening our understanding of the role of CRP, as an essential arm of the innate immune response and a central player in the pathogenesis of a range of inflammatory conditions, will be vitally important in translating potential anti-inflammatory, anti-CRP approaches to the clinic. However, to date, an important limitation in exploring the direct effects of CRP inhibition in these chronic diseases is the lack of suitable mouse models. Currently, the most widely used approach to study the role of CRP *in vivo* is the exogenous administration of pCRP to mice or rats prior to an acute inflammatory challenge. This is due to basic phylogenic differences in CRP biology between rodents and humans. For example, mice express very low levels of CRP, while rat CRP cannot activate complement (100, 101).

Therefore, while the administration of exogenous human CRP to rodents has afforded essential insights regarding CRP biology, it does not allow for *in vivo* mechanistic studies of CRP in chronic inflammatory conditions such as IRI, transplant rejection, atherosclerosis, or AMD. Thus, the development of novel animal models that express human CRP will allow more detailed studies regarding the precise mechanism of CRP dissociation in inflammation and its potential role as a novel therapeutic target.

## Author Contributions

JM, JK, SE, and KP conceived and co-wrote the manuscript. JL-S, DB, and LP co-wrote the manuscript.

## Conflict of Interest Statement

The authors declare that the research was conducted in the absence of any commercial or financial relationships that could be construed as a potential conflict of interest.
